# A Chinese cave links climate change, social impacts, and human adaptation over the last 500 years

**DOI:** 10.1038/srep12284

**Published:** 2015-08-13

**Authors:** Liangcheng Tan, Yanjun Cai, Zhisheng An, Hai Cheng, Chuan-Chou Shen, Sebastian F. M. Breitenbach, Yongli Gao, R. Lawrence Edwards, Haiwei Zhang, Yajuan Du

**Affiliations:** 1 State Key Laboratory of Loess and Quaternary Geology, Institute of Earth Environment, Chinese Academy of Sciences, Xi’an 710061, China; 2 Joint Center for Global Change Studies (JCGCS), Beijing 100875, China; 3 Institute of Global Environmental Change, Xi’an Jiaotong University, Xi’an 710054, China; 4 Department of Earth Sciences, University of Minnesota, Minneapolis 55455, USA; 5 Department of Geosciences, National Taiwan University, Taipei 106, Taiwan; 6 Department of Earth Sciences, University of Cambridge, Cambridge, CB2 3EQ, UK; 7 Center for Water Research, Department of Geological Sciences, University of Texas at San Antonio, San Antonio 78249, USA

## Abstract

The collapse of some pre-historical and historical cultures, including Chinese dynasties were presumably linked to widespread droughts, on the basis of synchronicities of societal crises and proxy-based climate events. Here, we present a comparison of ancient inscriptions in Dayu Cave from Qinling Mountains, central China, which described accurate times and detailed impacts of seven drought events during the period of 1520–1920 CE, with high-resolution speleothem records from the same cave. The comparable results provide unique and robust tests on relationships among speleothem δ^18^O changes, drought events, and societal unrest. With direct historical evidences, our results suggest that droughts and even modest events interrupting otherwise wet intervals can cause serious social crises. Modeling results of speleothem δ^18^O series suggest that future precipitation in central China may be below the average of the past 500 years. As Qinling Mountain is the main recharge area of two large water transfer projects and habitats of many endangered species, it is imperative to explore an adaptive strategy for the decline in precipitation and/or drought events.

In recent years, increasing attention is paid to the impact of climate change and adaptation strategies^1^,^2^. It is evident that climate change could pose critical impact on ecosystems and society. For instance, rainfall amount deviations directly affect agricultural crop yields, forest advance and retreat, and human health^3^,^4^,^5^. In particular, drought events have widely occurred on various timescales and in turn played an important role in changing social stability and human welfare during pre-historical and historical times^6^,^7^,^8^,^9^. Collapses of many ancient civilizations, such as the Neolithic culture in north central China^10^, the Akkadian Empire^6^, pyramid-constructing Old Kingdom civilization of Egypt^9^ and classic Maya^11^,^12^, have all been linked to intense droughts during mid- and late Holocene, on the basis of apparent synchronicities between proxy-inferred drought events and historically documented societal crises.

In the past decade, stalagmite δ^18^O records from China have characterized many aspects of the Asian monsoon variability over the past 500 ka years on centennial to orbital scales^13^,^14^,^15^. These records also show a possible linkage between climate change and the demise of several Chinese dynasties during the last 1800 years, such as Tang, Yuan and Ming Dynasties^16^,^17^. However, the relationship between Chinese stalagmite δ^18^O variations, monsoon climate change, and social crises is still in dispute^18^, and more evidence is required to evaluate the impact of past climate change.

Dayu Cave (33°08′ N, 106°18′ E, 870 m a. s. l.) is located on the southern slope of the Qinling Mountains, central China, and is more than 2 km long^19^ (Fig. 1). The cave has a high relative humidity (>97%) and high CO_2_ concentration (1600 ppm in the central pathway on 22 September, 2009). The cave temperature is 13.0 °C, consistent with the local annual mean temperature (12.9 °C). Climate in this region is dominated by the Asian monsoon system, with a mean annual rainfall of 1100 mm of which >70% are received during the summer monsoon months (June-October) (Fig. S1). Monitoring of precipitation above the cave between June 2010 and May 2011 shows that the δ^18^O of precipitation is lower during summer monsoon seasons (Fig. S2). Water balance analysis at the cave site indicates that most water surplus occurs between July and October (Fig. S1). Recharge of the aquifer thus occurs mainly during summer monsoon seasons. Spatial correlation analysis indicates that precipitation changes at the cave are positively correlated with those in central China (Fig. S3).

## Results

**Figure 2:**
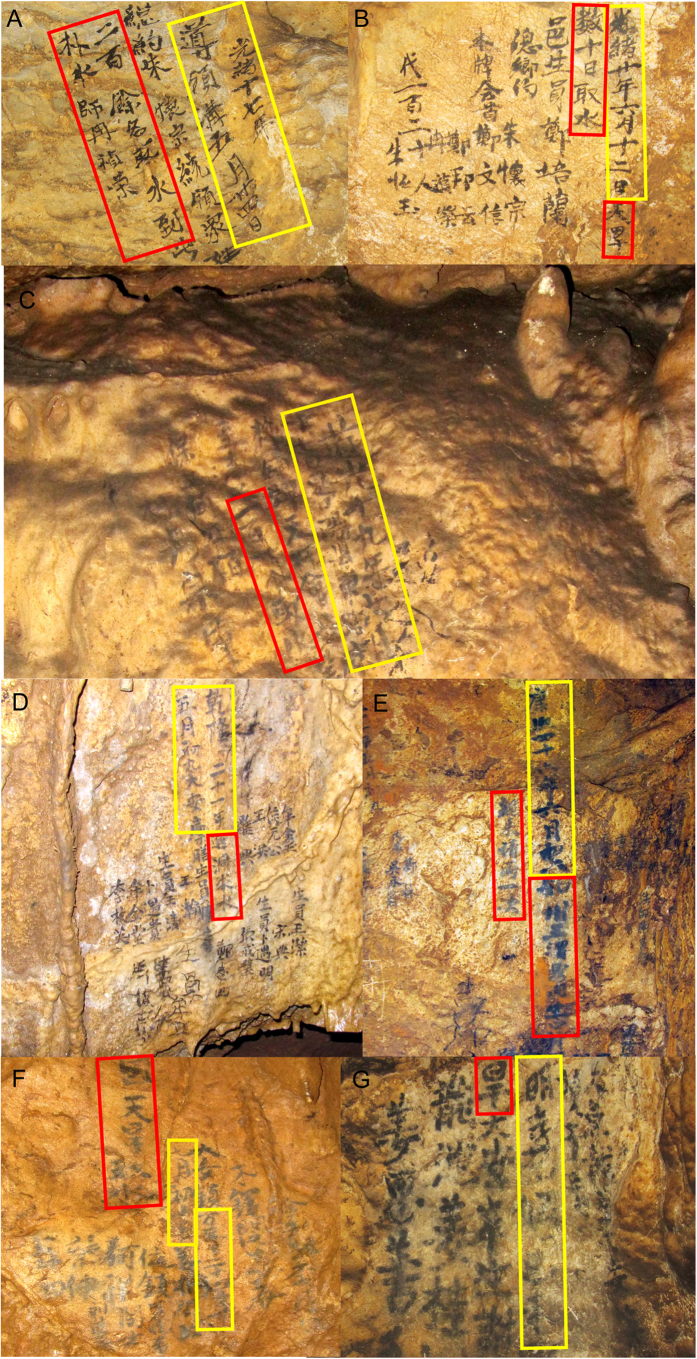
Photos of ancient inscriptions inside Dayu Cave, which recorded seven drought events. The yellow and red panels mark dates and the descriptions of drought events, respectively. All photos were taken in Dayu Cave by L Tan.

Many ancient inscriptions were disclosed in Dayu Cave, which indicate that local ancient people visited the cave frequently, at least 70 times during 1520–1920 CE. According to the inscriptions, seven major drought events were clearly described , occurring in 1528 CE, 1596 CE, 1707 CE, 1756 CE, 1839CE, 1891 CE and 1894 CE (Fig. 2), respectively. These inscriptions described many details of the droughts (Table 1). For example, one of them (Fig. 2A) stated: “On May 24th, 17th year of the Emperor Guangxu period, Qing Dynasty [the traditional Chinese calendar, equivalent to June 30th, 1891 CE], the local mayor, Huaizong Zhu led more than 200 people into the cave to retrieve water. A fortuneteller named Zhenrong Ran prayed for rain during a ceremony”. Three years later in 1894 CE (June 12th, 20th year of the Emperor Guangxu period, Qing Dynasty), another drought event occurred. The same mayor and fortuneteller again led more than 120 people into the cave to collect water (Fig. 2B). Another inscription indicated that “On June 8th, 46th year of the Emperor Kangxi period, Qing Dynasty [July 7th, 1707 CE], the governor of Ningqiang district came to the cave to pray for rain”.

**Figure 3:**
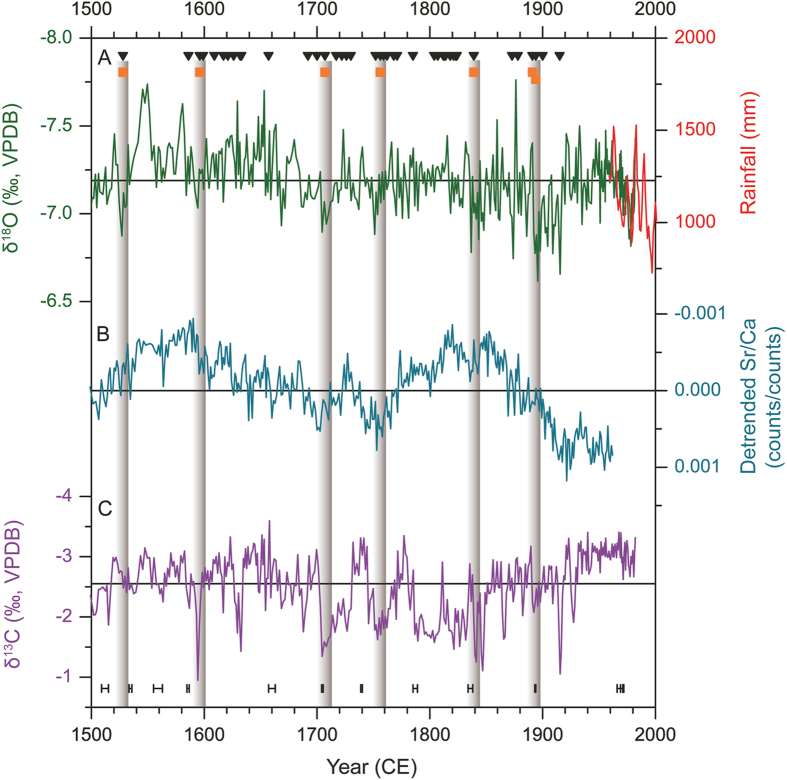
Comparison of drought events recorded in the inscriptions with speleothem δ^18^O, δ^13^C and Sr/Ca records in Dayu Cave during the last 500 years. The black triangles indicate 70 visits recorded in the cave, with some occurred in the same year. The orange squares indicate seven historical drought events occurred in 1528 CE, 1596 CE, 1707 CE, 1756 CE, 1839CE, 1891 CE and 1894 CE, respectively. (**A**) δ^18^O record of DY1 (dark green). The red line represents annual rainfall amount record from the Ningqiang meteorological station, 38 km south of Dayu Cave, during the period 1957–2000 CE, with a 3-year moving average. (**B**) Detrended Sr/Ca record of DY1(light blue); (**C**) δ^13^C record of DY1 (purple). Black vertical bars show locations of ^230^Th dates, with errors of ±0.4 to ±4 years. The straight lines in panel A and C indicate the average δ^18^O (−7.19‰) and δ^13^C (−2.54‰) values of the entire series, respectively.

The seven drought events described in the inscriptions are notably reflected in the stable isotopic and trace elemental records of a stalagmite DY1 from the Dayu cave (Fig. 3). DY1 was collected about 1 km from the cave entrance, covering the period from ca. 1265 to 1982 CE continuously (Fig. S4, Table S1). The initial low-resolution δ^18^O results from DY1 stalagmite were reported in 2009^19^. Here we built a more solid age model with additional six ^230^Th dates and a higher resolved (~1.3 yrs) stable isotopic and trace elemental profiles capturing annual δ^18^O, δ^13^C (Table S2) and Sr/Ca ratio variations during the last 500 years.

“Hendy test”^20^ results show that both the δ^18^O and δ^13^C remain constant along growth layers of DY1 (Fig. S5). Some limitations of “Hendy test” were reported. For example, the isotopic equilibrium could theoretically occur in the center of the speleothem at the same time that kinetic fractionation occurs at the flanks^21^. However, the stalagmite was most likely deposited at isotopic equilibrium conditions, if the isotopic values remain constant along growth layers. In addition, the DY1 δ^18^O record is similar to a calcite stalagmite SF1 (r = 0.21, N = 393, p < 0.001) from the Buddha Cave in the southern Qinling Mountains^22^, 300 km northeast of the Dayu Cave, on decadal scale, with different mineral compositions and amplitudes of δ^18^O variations (Fig. S6). The correlation coefficient is not very high, mainly because of the uncertainties of the chronology of SF1, which was based on two average growth rates during the last 500 years. The “Hendy test”^20^ and “Replication test”^21^ indicate that DY1 deposited under conditions close to isotopic equilibrium, and its δ^18^O and δ^13^C variations can be interpreted as proxies primarily reflecting climate and environment variations.

There is a significant negative correlation between the DY1 δ^18^O and the annual rainfall during the period between ~1957 and 1982 CE (r = −0.44, N = 24, p < 0.05). The correlation coefficient is not very high which may be ascribed to the “Smoothing effect” of the δ^18^O in drip water. The intra/inter- annual mixture of “fresh water” and “old water” may occurred in the karst aquifer^23^ of Dayu Cave, because of the thick epikarst zone (~80m). The age uncertainties may also play a role. The age model between the section of 1950–1982 CE of DY1 was built by an average growth rate of 0.197 mm/yr based on two ^230^Th dates at 1970 ± 1 and 1894 ± 0.4 CE. Because of possible variations in growth rate, the use of the average value may cause age uncertainties in given subsamples. Nevertheless, the δ^18^O sequence agrees with the observed annual and monsoon rainfall amount on long-term trend, with higher δ^18^O values corresponding to reduced precipitation, and vice versa (Fig. S7). During the last 500 years, the notable positive excursions in δ^18^O coincided with the droughts documented in the inscriptions, corroborating a generally inverse relationship between rainfall amount (mainly from summer monsoon) and speleothem δ^18^O in this region.

The δ^13^C values^24^ are relatively higher during each drought event in the last 500 years too (Fig. 3). In fact, there is a significant positive correlation (r = 0.37, P < 0.01, N = 393) between the δ^13^C and the δ^18^O records of DY-1 during 1500–1982 CE (Fig. S8). Speleothem δ^13^C values have bedrock, atmospheric, and soil gas sources^25^. As a result, many factors may affect the speleothem δ^13^C variations, including: (1) the fracture of epikarst zone and difference of lattice work in vadose zone (open/closed systems), (2) the extent of dissolution of the host rock, (3) the overlying vegetation types and density, (4) the microbial activity in the overlying soil, (5) prior precipitation of calcite (PCP) in the epikarst zone, (6) and the evaporation and degassing of drip water^25^,^26^,^27^. The constant temperature inside Dayu Cave suggests it is a closed system. Factors (3) and (4) are related to the vegetation change and climatic conditions. On decadal- to annual- time scales, cold and dry climate could reduce the vegetation cover and microbial activity, and result in higher δ^13^C values in speleothems. Factors (2) and (5) are related to hydrogeochemical processes in the epikarst zone and affected by climatic conditions. The increased residence time of the seepage water during drier conditions may allow more bedrock to be dissolved, favor PCP in the unsaturated zone, resulting in higher δ^13^C values in speleothem^25^,^26^,^27^. In addition, dry condition may enhance the evaporation and CO_2_ degassing of drip water^28^, and cause higher δ^13^C of speleothems in Dayu Cave.

As shown in Fig. 3, the droughts in 1596 CE, 1707 CE, 1756 CE, and 1839CE corresponded well with elevated Sr/Ca ratios. The other three droughts in 1528 CE , 1891 CE, and 1894 CE are also comparable with increased Sr/Ca ratios, considering age differences caused by different sampling intervals and paths. As discussed before, drier conditions could promote longer water residence times in the epikarst, decreased drip rates, and enhanced CO_2_ degasing into air voids within the unwetted epikarst. These conditions lead to Sr/Ca ratios higher than congruent bedrock dissolution due to preferential removal of Ca during PCP and increase the Sr/Ca ratio in speleothem^29^,^30^,^31^. The positive correlation between δ^18^O and Sr/Ca records of DY1 (r = 0.22, P < 0.01, N = 393) further confirm the observed inverse relationship between speleothem δ^18^O and rainfall amount in this region.

In summary, Dayu Cave provides for the first time an *in situ* comparison between historical drought events and speleothem records from the same cave. The in-phase variations in speleothem δ^18^O, δ^13^C and Sr/Ca during droughts in the last 500 years demonstrate a convincing anti-correlation between rainfall amount and speleothem δ^18^O in this region^32^.

## Discussion

### Impacts of climate change on local society

Historical documents show that drought events recorded in Dayu Cave caused serious social problems. For example, the drought of 1528 CE led to “a big starvation and cannibalism”^33^ around the Qinling Mountain region, from southern and central Shaanxi Province to eastern Gansu Province^34^. Droughts in the 1890s also caused severe starvation and triggered local social instability, which eventually resulted in a fierce conflict between government and civilians in 1900 CE^35^. A recent study suggested that the collapse of classic Maya civilization was caused by modest reduction in precipitation^11^,^12^. Similarly, comparisons between stalagmite δ^18^O changes and historical records inside Dayu Cave (Fig. 3) also provide robust evidence that even modest droughts during relatively wet periods had serious societal consequences. As shown in Fig. 3, the drought around 1596 CE was not very severe in comparison with others during the last 500 years in the Dayu δ^18^O record. However, in the context of the overall wet climate during 1530–1685 CE, the multi-year drought appears to be unusual and caused local societal unrest. The inscriptions (Fig. S9) describe the event as “mountains are crying due to drought”, and local people “came to the cave to get water” in July and August when the summer monsoon is presumably the strongest. The δ^13^C value of the stalagmite also reaches the highest value in the last 500 years in the drought in 1596 CE^25^.

As described in the inscriptions, in an attempt to adapt to droughts, people in the area came to the cave to obtain water and pray for rain. Likewise, during recent droughts in southwestern China, karst groundwater became a very important water source for local people. This demonstrates a common human adaptation to such climatic changes under similar conditions.

### Future climate change in southern Qinling Mountain region

**Figure 4:**
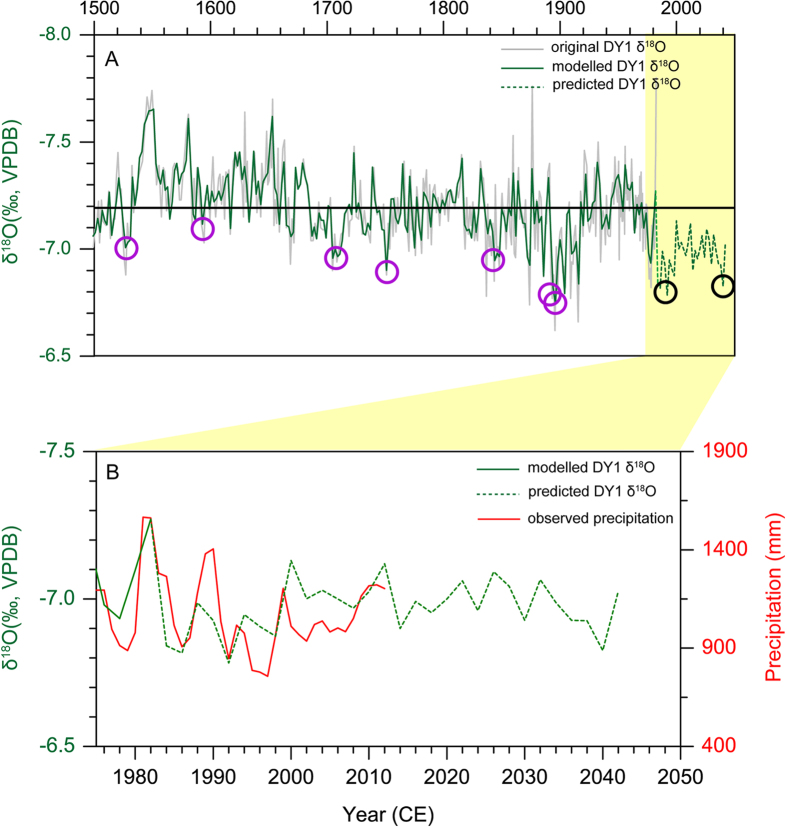
(**A**) Comparison between the original DY1 δ^18^O series (grey line) and the modeled (solid green line, with a 2-year resolution) and predicted stalagmite δ^18^O series (broken green line). The predicted interval is 1982–2042 CE. The straight black line indicates the average δ^18^O value during the last 500 years. The purple circles represent seven drought events recorded in the cave inscriptions during the period of 1520–1920 CE. The black circles denote two droughts predicted by the model, being occurred in the 1990 s and the late 2030 s, respectively. (**B**) Comparison between the predicted stalagmite δ^18^O (Broken green line) and annual precipitation records (red, with a 2-year moving average) in the Dayu Cave area.

A time domain combined model^36^ was used here to evaluate potential future precipitation changes in the area on the basis of the Dayu δ^18^O series. The modeled δ^18^O series fits the original series before 1982 CE, and is then extrapolated for 60 years to 2042 CE (Fig. 4A). Strong coherence between the predicted δ^18^O series and the observed precipitation variations in the period of 1982–2012 CE further validate this approach (Fig. 4B). According to our predicted δ^18^O changes, precipitation between 1982 and 2042 CE will likely fall below the average over the past 500 years in central China. Two droughts, comparable with historical droughts, appear in the model: the 1990s and the late 2030s. Instrumental data confirm the first drought event in the 1990 s (Fig. 4B).

**Figure 5:**
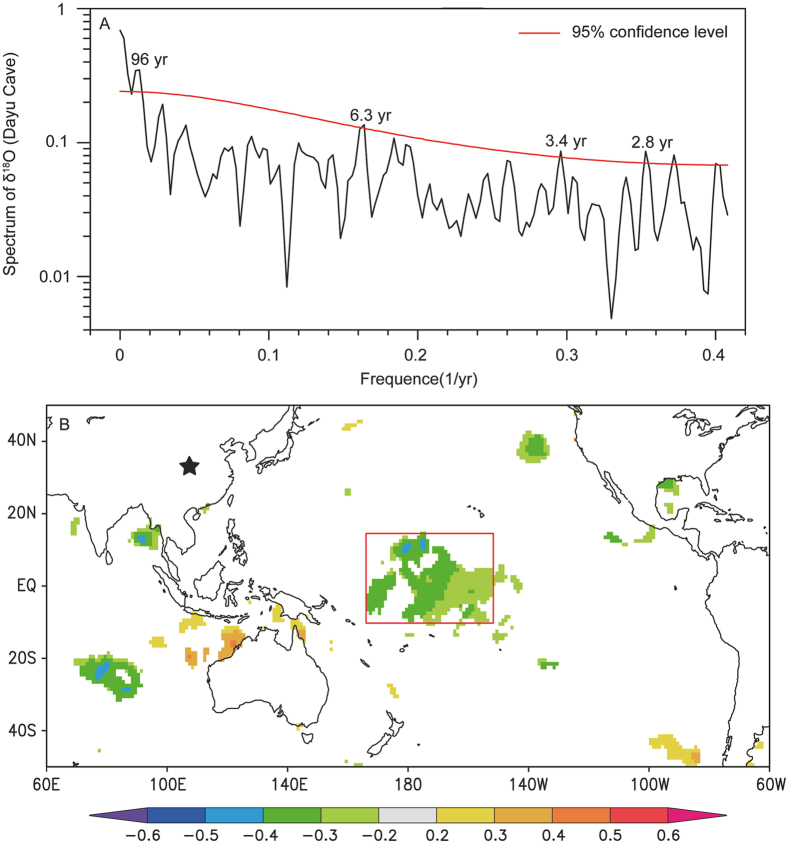
(**A**) Spectrum analysis results of the DY1 δ^18^O record over the last 500 years. Numbers within the graph indicate significant periodicities of 96, 6.3, 3.4 and 2.8 years that are above the 95% confidence level. The analysis was performed using Redfit 35^40^. The 6.3-, 3.4- and 2.8-year periodicities correspond to the 2–7 year cycle of El Nińo–Southern Oscillation^41^. (**B**) Spatial correlation between the CRU gridded annual precipitation around the Dayu Cave area (33–33.5° N, 106–106.5° E) and the HadlSST1 SST datasets between 1960 and 2009 CE. The star indicates the location of Dayu Cave. The red box denotes a significant negative correlation region in cetral Pacific. The scale bar at the bottom shows the correlation coefficients represented by different colors. The analysis was performed by the KNMI Climate Explorer^42^.

Spectral analysis of the Dayu record yields significant periodicities at 96, 6.3, 3.4 and 2.8 years, with the 2.8, 3.4, and 6.3 year periods corresponding to the El Nińo–Southern Oscillation (ENSO) cycle (Fig. 5A). Spatial correlation analysis also indicates that the precipitation in Dayu Cave region anti-correlates with the sea surface temperature (SST) of Niño 4 region during the period of 1960–2009 CE (Fig. 5B). Results of climate model simulations suggest that the tropical Pacific SST gradient decreases under conditions of global warming, resembling El Nińo-like SST patterns^37^,^38^. If the current warming continues, precipitation may decrease in the cave region, which is consistent with our prediction.

Precipitation in the southern Qinling Mountain region is the main recharge of Danjiangkou Reservoir, which supplies water to the middle route of South-to-North Water Transfer Project in China. Recently, another large Hanjiang-to-Weihe River Water Transfer Project is under construction, and its water source is similarly recharged by precipitation in the southern Qinling Mountain region. In addition, Qinling Mountains are important refugia for many rare and endangered species including giant pandas (*Ailuropoda melanoleuca*)^39^. It is therefore crucial to explore an adaptive strategy to prepare for the possible future decline in precipitation and/or drought events in the region.

## Methods

### ^230^Th dating

Stalagmite DY1 was cut into halves along the growth axis and polished. Subsamples for ^230^Th dating were obtained by drilling along the growth axis of the stalagmites with a hand-held carbide dental drill. The chemical procedure used to separate uranium and thorium followed those described in *ref. S1*. Measurements of uranium and thorium were performed on inductively coupled plasma mass spectrometers (ICPMS), Thermo-Finnigan ELEMENT and Thermo Fisher NEPTUNE, following procedures described in *ref. S2* and *ref. S3*, respectively. Corrections for initial ^230^Th were made assuming an initial ^230^Th/^232^Th atomic ratio of 4.4 ± 2.2 × 10^−6^. A total of 15 ^230^Th dates were obtained for DY1 (Table S1). Two-sigma date errors are less than 4 years for 13 layers. Linear interpolations between ^230^Th dates were used to establish the chronology (Fig. S4).

### Stable isotope and trace elements analyses

We performed stable isotope analyses (δ^18^O and δ^13^C) for DY1 at intervals from 100 μm to 250 μm, depending on growth rate. All subsamples were analyzed on an on-line, automated carbonate preparation system (Kiel III) linked Finnigan MAT-252 gas source mass spectrometer (Table S2). International standard NBS19 and inter-laboratory standard TTB1 were measured every 10–15 samples. Arbitrarily selected duplicates were conducted to check the homogeneity and reproducibility. Stable isotopic values reported are relative to the Vienna PeeDee Belemnite (VPDB) standard. The standard results show that precisions of δ^18^O and δ^13^C analysis are better than 0.1‰ (2σ). Sr and Ca counts of the stalagmite were measured using the Itrax core scanner at the First Institute of Oceanography, State Oceanic Administration at 0.1 mm resolution.

### The prediction model

The DY1 δ^18^O series was decomposed to determine the significant harmonics and their corresponding periodicities (*ref. S4*). Based on the cumulative variance contribution of ~90%, there were 82 significant harmonics. We then used an Auto Regressive And Moving Average [ARMA(p, q)] model to simulate the residual errors of the δ^18^O series, which were produced by subtracting period terms from the original series. According to the Bayesian information criterion, the degrees *p* and *q* of the ARMA model were selected as 2 and 3, respectively. By combining the periodic terms and the results of ARMA(p, q) model, the prediction model of the δ^18^O series was established^36^.

## Additional Information

**How to cite this article**: Tan, L. *et al.* A Chinese cave links climate change, social impacts, and human adaptation over the last 500 years. *Sci. Rep.* **5**, 12284; doi: 10.1038/srep12284 (2015).

## Supplementary Material

Supplementary Information

## Figures and Tables

**Figure 1:**
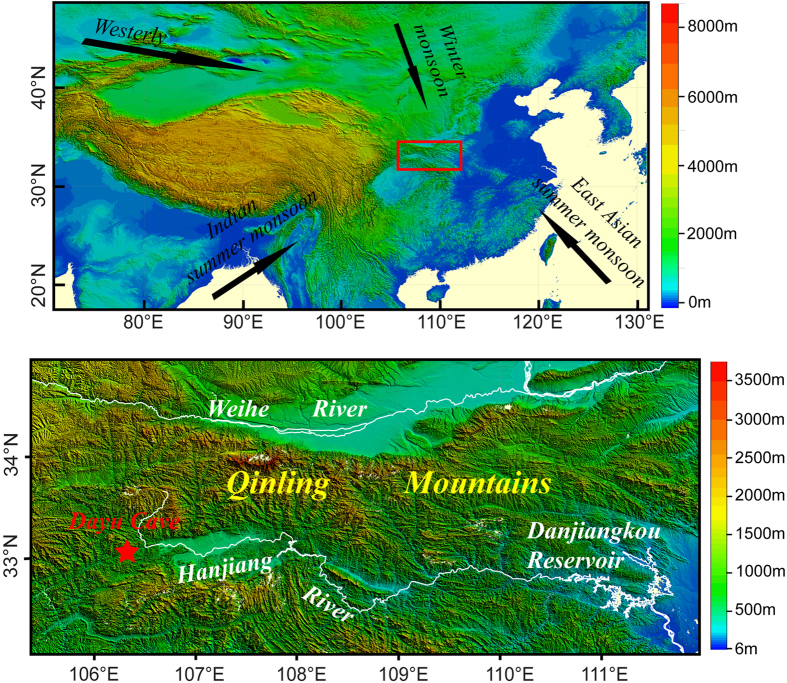
Location of Dayu Cave. The upper panel is an overview topographic map showing the study region (red rectangle). Black arrows denote the directions of the East Asian summer monsoon, Indian summer monsoon, East Asian winter monsoon, and Westerly, which affect the climate in China. The lower panel is an enlarged map showing the location of Dayu Cave (33°08′ N, 106°18′ E, 870 m a. s. l.) in southern Qinling Mountains. The Hanjiang and Weihe River, as well as the Danjiangkou Reservoir are also shown. GTOPO30 data distributed by U.S. Geological Survey’s EROS (Earth Resources Observation and Science) Data Center were used to plot the topographic map with the software of Global Mapper 9.

**Table 1:** Seven drought events recorded in the ancient inscriptions inside Dayu Cave during the period of 1500–1920 CE.

**Droughts**	**Solar calendar dates**	**Descriptions**
1	1528 CE	Drought occurred in 7th year of the Emperor Jiajing period, Ming Dynasty (the traditional Chinese calendar-authors). Gui Jiang and Sishan Jiang came to Da’an town (the town where Dayu Cave is located-authors) to acknowledge the Dragon Lake inside in Dayu Cave.
2	July 27th, 1596 CE	On July 3rd, 24th year of the Emperor Wanli period, Ming Dynasty, local people came to the cave to get water because of the big drought (their name are omitted here-authors).
3	July 7th, 1707 CE	On June 8th, 46th year of the Emperor Kangxi period, Qing Dynasty, the governor of Ningqiang district came to the cave to pray for rain.
4	June 6th, 1756 CE	On May 9th, 21th year of the Emperor Qianlong period, Qing Dynasty, 17 scholars came to the cave to pray for rain (their name are omitted here-authors)
5	July 27th, 1839 CE	On June 17th, 19th year of the Emperor Daoguang period, Qing Dynasty, 120 persons from Lueyang county (a county in the north of Dayu Cave-authors) came to the cave to get water (other details are illegible-authors).
6	July 30th, 1891 CE	On May 24th, 17th year of the Emperor Guangxu period, Qing Dynasty, the local mayor, Huaizong Zhu led more than 200 people into the cave to get water. A fortuneteller named Zhenrong Ran prayed for rain during a ceremony.
7	June 14th, 1894 CE	Drought lasted for more than one month. On June 12th, 20th year of the Emperor Guangxu period, Qing Dynasty, scholar Peilan Zheng, mayor Huaizong Zhu, heads of the clan Wenxin Zheng and Bangyun Zheng, and Zhenrong Ran, Hengyu Zhu, led more than 120 persons to the cave to get water.
