# Supplementary material for: A Chinese cave links climate change, social impacts, and human adaptation over the last 500 years
Source: Sci Rep. 2015 Aug 13;5:12284. doi: 10.1038/srep12284 (PMC4535275; doi:10.1038/srep12284)
Supplement: Supplementary Information [file srep12284-s1.doc]

**Supplementary Materials for**

**A Chinese cave links climate change, social impacts and human adaptation over the last 500 years**

Liangcheng Tan^1*^, Yanjun Cai^1^, Zhisheng An^1^, Hai Cheng^2,3^, Chuan-Chou Shen^4^,

Sebastian F.M. Breitenbach^5^, Yongli Gao^6^, R. Lawrence Edwards^3^, Haiwei Zhang^2^ & Yajuan Du^1^

1. State Key Laboratory of Loess and Quaternary Geology, Institute of Earth Environment, Chinese Academy of Sciences, Xi’an 710061, China;

2. Institute of Global Environmental Change, Xi’an Jiaotong University, Xi’an 710054, China;

3. Department of Earth Sciences, University of Minnesota, Minneapolis 55455, USA;

4. Department of Geosciences, National Taiwan University, Taipei 106, Taiwan;

5. Geological Institute, Department of Earth Sciences, ETH Zürich, Zürich 8092, Switzerland;

6. Center for Water Research, Department of Geological Sciences, University of Texas at San Antonio, San Antonio 78249, USA

*To whom correspondence should be addressed: E-mail: [tanlch@ieecas.cn](mailto:tanlch@ieecas.cn)

**Contents of this file**

Figures S1 to S9

Tables S1 and S2

**Supplementary Figures**


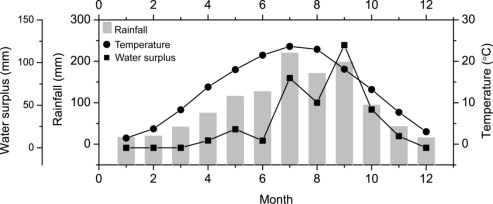


**Figure S1.** Monthly rainfall (gray bars), temperature (circles), and water surplus (squares) at Ningqiang station (data: from 1957 to 2009 CE).The water balance was calculated by the Thornthwaite evapotranspiration model (*refs. S5, S6*).


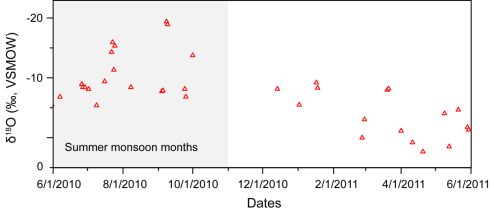


**Figure S2.** The δ^18^O values of precipitation at the Dayu Cave site between June 2010 and May 2011.


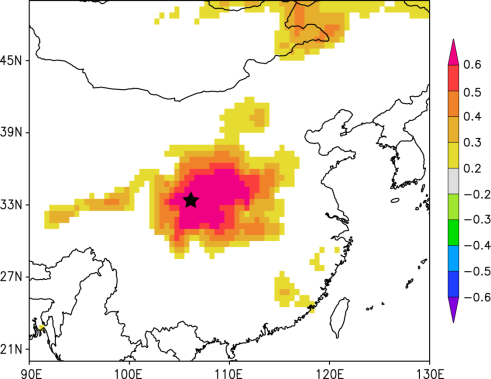


**Figure S3.** Spatial correlation between the CRU gridded annual precipitation around the Dayu Cave area (33-33.5° N, 106-106.5° E) and the CRU TS3.10.01 precipitation grid datasets between 1960 and 2009 CE. The star indicate the location of Dayu Cave. The scale on the right shows the correlation coefficients represented by different colors. It shows that the annual precipitation changed synchronously over central China during this period. The analysis was performed by the KNMI Climate Explorer^42^.


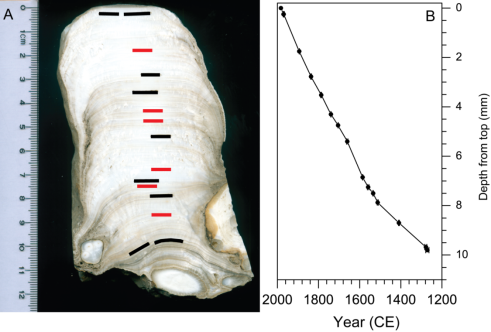


**Figure S4.** (A) A photograph of polished surface, and (B) ^230^Th age model for the stalagmite DY1. The black lines in panel A indicate the locations of nine ^230^Th dating subsamples, including two replicates, measured by Thermo-Finnigan ELEMENT^19^. The red lines indicate the locations of six more ^230^Th dating subsamples measured by Thermo Fisher NEPTUNE(*ref. S3*). The chronology was constructed by linear interpolation between ^230^Th dates. It suggests that the growth rate of DY1 varied from 0.074 to 0.196 mm/yr.


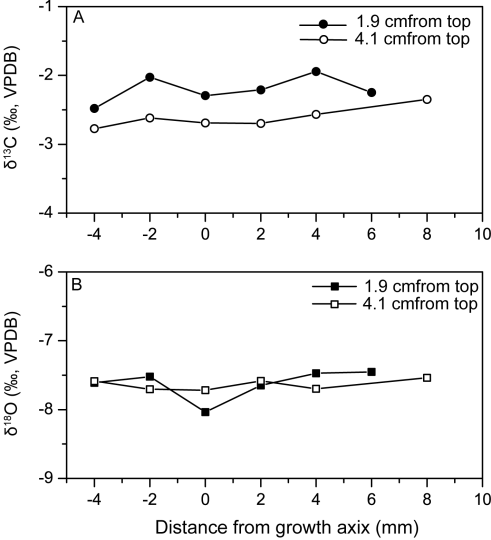


**Figure S5.** Results of the Hendy test on the (A) δ^13^C and (B) δ^18^O records from two horizons of the stalagmite DY1. The δ^18^O and δ^13^C remained constant along the growth layers, indicating that it was deposited under equilibrium conditions of isotopic fractionation.


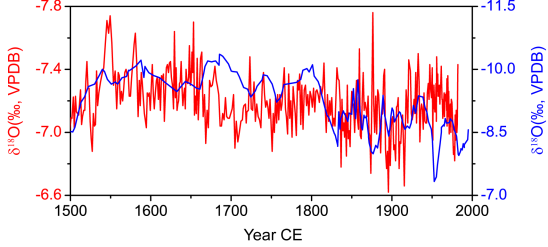


**Figure S6.** Comparison between the DY1 δ^18^O (red) record and a calcite stalagmite SF1 (blue) from the Buddha Cave in the southern Qinling Mountains^22^, 300 km northeast of the Dayu Cave. The two records are similar in general(r= 0.21, N=393, p< 0.001), although with different mineral compositions and amplitudes. The discrepancy observed may be the result of dating errors and the different depth-age models used. The chronology of SF1 is based on an average growth rate of 0.083 mm/yr for the last 150 years and an average growth rate of 0.0163 mm/yr before that. The former was determined by counting the annual laminations and ^210^Pb dating, and the latter was determined from TIMS ^230^Th dating at 3100 years with a dating error of 50 years^22^.


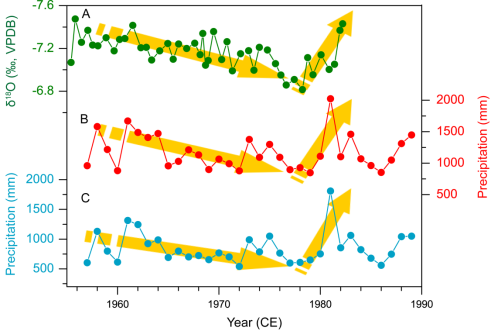


**Figure S7.** Comparison of the δ^18^O record of DY1(A) to annual (B) and monsoon precipitation (C) recorded at Ningqiang meteorological station, 38 km to the south of Dayu Cave. The stalagmite δ^18^O signal largely anti-correlates (r = -0.44, N = 24, p < 0.05) with precipitation (mainly monsoon precipitation) on annual- to decadal- scales. The dark yellow arrows represent variation trends of the three series.


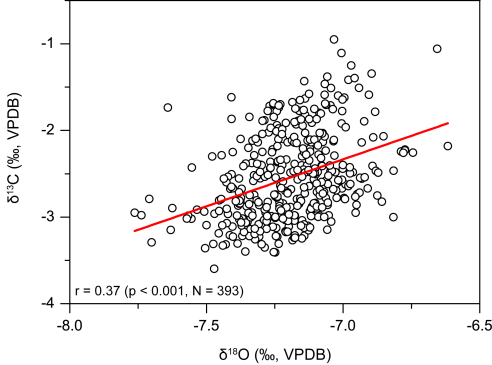


**Figure S8.** Correlation of δ^18^O vs δ^13^C in DY1. There is a significant positive correlation (r = 0.37, p < 0.01, N = 393) between δ^18^O and δ^13^C records, which suggests that reduced precipitation could also produce heavier δ^13^C values in speleothem by reducing vegetation cover, dissolving more bedrock and favor PCP in the unsaturated zone above the cave^25^.


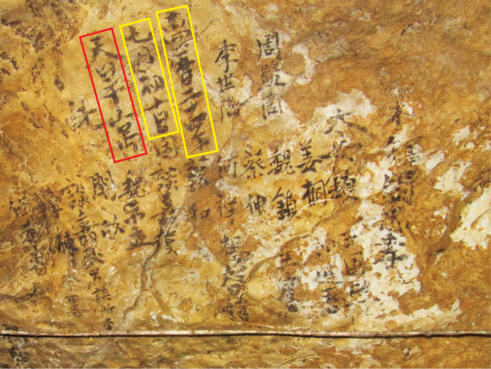


**Figure S9.** Another inscription inside in Dayu Cave described the drought occurred in 1596 CE. It told that “On July 10th, 24th year of the Emperor Wanli period, Ming Dynasty (August 3rd, 1596 CE), mountains are crying due to drought (the visitors’ names are omitted-authors)”.

**Supplementary references**

S1. Edwards RL, Chen JH, Wasserburg GJ. ^238^U-^234^U-^230^Th-^232^Th systematics and the precise measurement of time over the past 500000 years. *Earth Planet. Sci. Lett.* 1987, 81(2-3):175-192.

S2. Shen C-C, Lawrence Edwards R, Cheng H, Dorale JA, Thomas RB, Bradley Moran S, et al. Uranium and thorium isotopic and concentration measurements by magnetic sector inductively coupled plasma mass spectrometry. *Chem. Geol.* 2002, 185(3):165-178.

S3. Shen C-C, Wu C-C, Cheng H, Lawrence Edwards R, Hsieh Y-T, Gallet S, et al. High-precision and high-resolution carbonate ^230^Th dating by MC-ICP-MS with SEM protocols. *Geochim. Cosmochim. Acta* 2012, 99:71-86.

S4. Yang W, Gu L. *The time series analysis and dynamic data modelling*. Beijing Institute of Technology Press: Beijing, 1988.

S5. McCabe, G. J., and S. L. Markstrom, *A monthly water-balance driven by a graphical user interface: US Geological Survey Open-File report 2007-1088*, 2007, 6 pp.

S6. Thornthwaite, C. W., An approach toward a rational classification of climate, *Geogr. Rev.* 1948, *38*, 55-94.

**Supplementary Tables**

Table S1. ^230^Th dating results of stalagmite DY1 from the Dayu Cave

| **Sample**  **ID** | **Depth**  **(mm)** | **^238^U**  **(ppb)** | **^232^Th**  **(ppt)** | **^230^Th / ^232^Th**  **(ppm)** | **δ^234^U**  **(measured)** | **^230^Th / ^238^U**  **(activity)** | **^230^Th Age (yr)**  **(uncorrected)** | **^230^Th Age (yr CE)**  **(corrected )** | **δ^234^U_Initial_**  **(corrected)** |
| --- | --- | --- | --- | --- | --- | --- | --- | --- | --- |
| DY1-8 | 98 | 20378±195 | 1364±11 | 4228±36 | 1551±9 | 0.01714±0.00017 | 735±8 | **1271±8** | 1554±9 |
| DY1-7 | 96.75 | 15556±23 | 1537±21 | 2841±42 | 1555±3 | 0.01700±0.00012 | 729±5 | **1278±5** | 1558±3 |
| DY1-6A* | 87 | 11158±18 | 311±5 | 8393±134 | 1566±4 | 0.01419±0.00004 | 604±2 | **1409±2** | 1569±4 |
| DY1-6 | 78.75 | 21893±46 | 927±16 | 4527±81 | 1570±4 | 0.01161±0.00008 | 494±3 | **1512±3** | 1572±4 |
| DY1-5A* | 75 | 13671±21 | 254±5 | 9900±187 | 1544±4 | 0.01115±0.00002 | 479±1 | **1535±1** | 1547±4 |
| DY1-5 | 72.5 | 12398±86 | 384±11 | 5523±158 | 1536±6 | 0.01036±0.00009 | 447±4 | **1559±4** | 1538±6 |
| DY1-4A* | 68.5 | 11007±13 | 156±5 | 11538±364 | 1537±3 | 0.00993±0.00002 | 427±0.9 | **1586±0.9** | 1539±3 |
| DY1-4 | 54 | 17096±35 | 612±18 | 3716±113 | 1546±4 | 0.00806±0.00007 | 346±3 | **1660±3** | 1547±4 |
| DY1-3A* | 47.5 | 14777±17 | 344±5 | 5052±81 | 1525±3 | 0.00714±0.00001 | 309±0.7 | **1705±0.7** | 1527±3 |
| DY1-2A* | 43 | 13139±15 | 357±5 | 3842±57 | 1527±3 | 0.00634±0.00001 | 274±0.6 | **1739±0.7** | 1528±3 |
| DY1-3 | 35.25 | 17660±128 | 585±10 | 2528±46 | 1526±7 | 0.00507±0.00005 | 219±2 | **1787±2** | 1526±7 |
| DY1-2 | 27.75 | 21618±39 | 400±21 | 3504±186 | 1526±3 | 0.00393±0.00005 | 170±2 | **1836±2** | 1527±3 |
| DY1-1A* | 17.5 | 1309±13 | 225±4 | 2669±43 | 1536±2 | 0.00278±0.00001 | 120±0.4 | **1894±0.4** | 1536±2 |
| DY1-1a | 2.5 | 13190±85 | 309±10 | 592±24 | 1538±6 | 0.00084±0.00002 | 36±1 | **1970±1** | 1538±6 |
| DY1-1 | 2.5 | 12429±20 | 3247±24 | 59±4 | 1547±3 | 0.00093±0.00006 | 40±2 | **1969±3** | 1548±3 |

The errors are 2σ errors.

Subsamples without * were measured by Thermo-Finnigan ELEMENT in 2007 CE, and that with * were measured by Thermo Fisher NEPTUNE in 2013 CE.

Corrected ^230^Th ages assume the initial ^230^Th/^232^Th atomic ratio of 4.4±2.2×10^-6^. Those are the values for a material at secular equilibrium, with the bulk earth ^232^Th/^238^U value of 3.8. The errors are arbitrarily assumed to be 50%.

Table S2 Oxygen and carbon isotopic data of stalagmite DY1 from Dayu Cave. Depths are relative to the top (youngest surface) of the stalagmites and are measured along the growth axis. Ages are linearly interpolated between ^230^Th dates (see Table S1). Oxygen isotope ratios are expressed in *δ* notation, and the per mil derived from the Vienna Pee-Dee Belemnite standard.

| **Depth** | **Age** | **δ^18^O** | **δ^13^C** | **Depth** | **Age** | **δ^18^O** | **δ^13^C** | **Depth** | **Age** | **δ^18^O** | **δ^13^C** |
| --- | --- | --- | --- | --- | --- | --- | --- | --- | --- | --- | --- |
| **(mm)** | **(CE)** | **(VPDB, ‰)** | **(VPDB, ‰)** | **(mm)** | **(CE)** | **(VPDB, ‰)** | **(VPDB, ‰)** | **(mm)** | **(CE)** | **(VPDB, ‰)** | **(VPDB, ‰)** |
| 0.10 | 1982.2 | -7.43 | -3.32 | 3.80 | 1963.4 | -7.09 | -3.00 | 7.98 | 1942.1 | -7.06 | -2.78 |
| 0.16 | 1981.9 | -7.37 | -3.22 | 3.91 | 1962.8 | -7.21 | -3.16 | 8.19 | 1941.0 | -7.07 | -2.71 |
| 0.26 | 1981.4 | -7.05 | -2.98 | 4.01 | 1962.3 | -7.21 | -3.00 | 8.39 | 1940.0 | -7.25 | -3.41 |
| 0.36 | 1980.9 | -7.00 | -2.67 | 4.17 | 1961.5 | -7.42 | -3.21 | 8.59 | 1939.0 | -7.02 | -2.78 |
| 0.52 | 1980.1 | -7.14 | -3.10 | 4.32 | 1960.7 | -7.29 | -3.05 | 8.79 | 1938.0 | -7.14 | -3.09 |
| 0.68 | 1979.3 | -6.95 | -2.95 | 4.43 | 1960.2 | -7.28 | -2.95 | 8.99 | 1936.9 | -7.18 | -3.04 |
| 0.78 | 1978.8 | -7.11 | -2.88 | 4.53 | 1959.7 | -7.18 | -2.98 | 9.19 | 1935.9 | -7.14 | -3.12 |
| 0.89 | 1978.2 | -6.82 | -3.00 | 4.69 | 1958.9 | -7.30 | -3.09 | 9.40 | 1934.9 | -6.94 | -2.72 |
| 1.04 | 1977.4 | -6.91 | -2.67 | 4.84 | 1958.1 | -7.23 | -3.00 | 9.60 | 1933.9 | -7.32 | -3.26 |
| 1.20 | 1976.6 | -6.86 | -2.82 | 4.95 | 1957.5 | -7.23 | -2.99 | 9.80 | 1932.8 | -7.50 | -3.09 |
| 1.30 | 1976.1 | -6.95 | -2.86 | 5.04 | 1957.1 | -7.37 | -3.02 | 10.00 | 1931.8 | -7.34 | -3.22 |
| 1.41 | 1975.6 | -7.06 | -3.15 | 5.16 | 1956.4 | -7.26 | -3.11 | 10.21 | 1930.7 | -7.06 | -2.58 |
| 1.56 | 1974.8 | -7.19 | -3.27 | 5.28 | 1955.8 | -7.48 | -3.31 | 10.43 | 1929.6 | -7.38 | -2.82 |
| 1.72 | 1974.0 | -7.21 | -3.03 | 5.36 | 1955.4 | -7.07 | -3.00 | 10.64 | 1928.5 | -7.12 | -2.21 |
| 1.82 | 1973.4 | -7.00 | -2.78 | 5.44 | 1955.0 | -7.31 | -3.23 | 10.85 | 1927.5 | -7.21 | -1.91 |
| 1.93 | 1972.9 | -7.18 | -3.24 | 5.56 | 1954.4 | -7.19 | -3.04 | 11.07 | 1926.4 | -7.12 | -2.14 |
| 2.08 | 1972.1 | -7.15 | -3.04 | 5.69 | 1953.8 | -7.13 | -3.15 | 11.28 | 1925.3 | -7.08 | -2.41 |
| 2.24 | 1971.3 | -6.99 | -2.62 | 5.77 | 1953.4 | -7.13 | -3.10 | 11.50 | 1924.2 | -7.12 | -2.93 |
| 2.34 | 1970.8 | -7.26 | -2.99 | 5.85 | 1953.0 | -7.18 | -3.02 | 11.71 | 1923.1 | -7.39 | -3.31 |
| 2.45 | 1970.3 | -7.10 | -2.90 | 5.97 | 1952.3 | -7.30 | -3.11 | 11.92 | 1922.0 | -7.42 | -2.96 |
| 2.60 | 1969.5 | -7.36 | -3.40 | 6.09 | 1951.7 | -7.15 | -3.25 | 12.14 | 1920.9 | -7.46 | -3.19 |
| 2.71 | 1968.9 | -7.09 | -2.98 | 6.17 | 1951.3 | -7.25 | -3.01 | 12.35 | 1919.8 | -7.06 | -2.62 |
| 2.76 | 1968.7 | -7.04 | -3.05 | 6.25 | 1950.9 | -6.95 | -2.73 | 12.56 | 1918.7 | -7.19 | -2.68 |
| 2.81 | 1968.4 | -7.34 | -3.14 | 6.37 | 1950.3 | -7.41 | -3.11 | 12.78 | 1917.7 | -7.27 | -2.36 |
| 2.86 | 1968.1 | -7.14 | -3.22 | 6.57 | 1949.3 | -7.22 | -2.93 | 12.99 | 1916.6 | -6.98 | -1.42 |
| 2.97 | 1967.6 | -7.25 | -3.41 | 6.77 | 1948.2 | -7.28 | -3.14 | 13.21 | 1915.5 | -6.66 | -1.06 |
| 3.12 | 1966.8 | -7.20 | -3.11 | 6.98 | 1947.2 | -7.43 | -3.07 | 13.42 | 1914.4 | -6.92 | -1.90 |
| 3.28 | 1966.0 | -7.24 | -3.23 | 7.18 | 1946.2 | -7.21 | -3.17 | 13.63 | 1913.3 | -7.11 | -2.56 |
| 3.39 | 1965.5 | -7.10 | -2.99 | 7.38 | 1945.2 | -7.31 | -3.08 | 13.85 | 1912.2 | -7.06 | -2.83 |
| 3.49 | 1965.0 | -7.25 | -3.13 | 7.58 | 1944.1 | -7.25 | -3.22 | 14.06 | 1911.1 | -6.93 | -2.56 |
| 3.65 | 1964.2 | -7.18 | -3.21 | 7.78 | 1943.1 | -7.06 | -2.86 | 14.27 | 1910.0 | -6.94 | -2.55 |

Continue to next page

Table S2 (Cont.)

| **Depth** | **Age** | **δ^18^O** | **δ^13^C** | **Depth** | **Age** | **δ^18^O** | **δ^13^C** | **Depth** | **Age** | **δ^18^O** | **δ^13^C** |
| --- | --- | --- | --- | --- | --- | --- | --- | --- | --- | --- | --- |
| **(mm)** | **(CE)** | **(VPDB, ‰)** | **(VPDB, ‰)** | **(mm)** | **(CE)** | **(VPDB, ‰)** | **(VPDB, ‰)** | **(mm)** | **(CE)** | **(VPDB, ‰)** | **(VPDB, ‰)** |
| 14.49 | 1909.0 | -6.77 | -2.22 | 22.50 | 1865.5 | -7.14 | -1.63 | 30.00 | 1821.3 | -7.07 | -1.46 |
| 14.70 | 1907.9 | -7.22 | -2.84 | 22.71 | 1864.3 | -7.38 | -2.10 | 30.22 | 1819.9 | -7.24 | -1.74 |
| 14.91 | 1906.8 | -7.17 | -2.79 | 22.92 | 1863.1 | -7.22 | -2.92 | 30.44 | 1818.4 | -7.00 | -1.78 |
| 15.13 | 1905.7 | -7.16 | -2.85 | 23.14 | 1861.9 | -6.96 | -2.48 | 30.66 | 1817.0 | -7.16 | -1.77 |
| 15.35 | 1904.6 | -6.82 | -2.46 | 23.35 | 1860.7 | -6.98 | -2.49 | 30.88 | 1815.5 | -7.44 | -2.29 |
| 15.57 | 1903.4 | -6.92 | -2.54 | 23.56 | 1859.6 | -7.53 | -2.91 | 31.11 | 1814.1 | -7.29 | -1.88 |
| 15.79 | 1902.3 | -6.89 | -2.41 | 23.77 | 1858.4 | -7.02 | -2.57 | 31.33 | 1812.6 | -7.24 | -1.98 |
| 16.01 | 1901.2 | -7.10 | -2.84 | 23.98 | 1857.2 | -7.08 | -2.41 | 31.55 | 1811.2 | -7.29 | -2.91 |
| 16.23 | 1900.1 | -7.08 | -2.59 | 24.19 | 1856.0 | -7.33 | -2.95 | 31.77 | 1809.7 | -7.19 | -2.11 |
| 16.45 | 1899.0 | -7.03 | -2.48 | 24.41 | 1854.8 | -7.19 | -2.49 | 31.99 | 1808.3 | -7.24 | -1.78 |
| 16.67 | 1897.9 | -6.79 | -2.24 | 24.62 | 1853.6 | -7.00 | -2.40 | 32.21 | 1806.8 | -7.27 | -1.72 |
| 16.89 | 1896.7 | -7.01 | -2.58 | 24.83 | 1852.4 | -7.18 | -2.39 | 32.43 | 1805.4 | -7.26 | -1.70 |
| 17.11 | 1895.6 | -6.62 | -2.18 | 25.04 | 1851.2 | -7.17 | -2.57 | 32.65 | 1804.0 | -7.13 | -1.58 |
| 17.32 | 1894.5 | -6.88 | -2.47 | 25.24 | 1850.1 | -7.26 | -2.22 | 32.88 | 1802.5 | -7.28 | -1.77 |
| 17.54 | 1893.4 | -6.77 | -2.23 | 25.44 | 1849.0 | -7.13 | -2.40 | 33.10 | 1801.1 | -7.16 | -1.65 |
| 17.76 | 1892.1 | -6.89 | -2.08 | 25.63 | 1847.9 | -6.91 | -1.59 | 33.32 | 1799.6 | -7.23 | -1.70 |
| 17.98 | 1890.9 | -7.40 | -2.25 | 25.83 | 1846.8 | -7.01 | -1.11 | 33.54 | 1798.2 | -7.26 | -1.69 |
| 18.20 | 1889.7 | -7.36 | -3.17 | 26.03 | 1845.7 | -6.96 | -1.39 | 33.76 | 1796.7 | -7.11 | -1.75 |
| 18.42 | 1888.4 | -7.12 | -2.90 | 26.23 | 1844.5 | -7.30 | -2.40 | 33.98 | 1795.3 | -7.24 | -1.71 |
| 18.64 | 1887.2 | -7.11 | -2.84 | 26.43 | 1843.4 | -6.85 | -2.07 | 34.20 | 1793.8 | -7.17 | -1.93 |
| 18.86 | 1886.0 | -6.87 | -2.49 | 26.63 | 1842.3 | -7.12 | -2.25 | 34.42 | 1792.4 | -7.30 | -2.04 |
| 19.08 | 1884.7 | -7.10 | -2.88 | 26.83 | 1841.2 | -6.97 | -1.25 | 34.65 | 1790.9 | -7.27 | -1.97 |
| 19.30 | 1883.5 | -7.24 | -2.37 | 27.02 | 1840.1 | -7.06 | -1.38 | 34.87 | 1789.5 | -7.02 | -1.60 |
| 19.52 | 1882.3 | -7.27 | -2.02 | 27.22 | 1839.0 | -7.01 | -1.92 | 35.09 | 1788.0 | -7.30 | -1.89 |
| 19.74 | 1881.0 | -6.99 | -2.79 | 27.42 | 1837.9 | -7.21 | -2.70 | 35.31 | 1786.6 | -7.05 | -1.71 |
| 19.96 | 1879.8 | -7.23 | -2.83 | 27.62 | 1836.7 | -6.78 | -2.26 | 35.54 | 1785.2 | -7.37 | -2.78 |
| 20.17 | 1878.6 | -7.00 | -2.57 | 27.82 | 1835.6 | -7.19 | -2.88 | 35.76 | 1783.9 | -7.27 | -2.44 |
| 20.38 | 1877.4 | -7.18 | -2.98 | 28.02 | 1834.3 | -7.33 | -2.38 | 35.98 | 1782.5 | -7.33 | -2.12 |
| 20.59 | 1876.2 | -7.76 | -2.95 | 28.21 | 1833.0 | -7.34 | -1.84 | 36.21 | 1781.1 | -6.98 | -2.42 |
| 20.81 | 1875.0 | -7.17 | -2.43 | 28.41 | 1831.7 | -7.41 | -1.62 | 36.43 | 1779.8 | -7.12 | -2.99 |
| 21.02 | 1873.8 | -6.74 | -2.25 | 28.61 | 1830.4 | -7.24 | -1.94 | 36.65 | 1778.4 | -7.20 | -3.04 |
| 21.23 | 1872.7 | -6.98 | -2.64 | 28.81 | 1829.1 | -7.04 | -2.12 | 36.88 | 1777.0 | -7.29 | -3.35 |
| 21.44 | 1871.5 | -7.00 | -2.44 | 29.01 | 1827.8 | -7.09 | -1.80 | 37.10 | 1775.7 | -6.97 | -2.62 |
| 21.65 | 1870.3 | -7.23 | -2.87 | 29.21 | 1826.5 | -7.04 | -1.51 | 37.32 | 1774.3 | -7.20 | -2.72 |
| 21.86 | 1869.1 | -7.10 | -2.51 | 29.40 | 1825.2 | -7.22 | -2.10 | 37.54 | 1772.9 | -7.09 | -2.48 |
| 22.08 | 1867.9 | -7.15 | -2.40 | 29.60 | 1823.9 | -7.11 | -1.91 | 37.77 | 1771.6 | -7.37 | -3.10 |
| 22.29 | 1866.7 | -7.19 | -1.67 | 29.80 | 1822.6 | -7.20 | -2.14 | 37.99 | 1770.2 | -6.99 | -1.96 |

Continue to next page

Table S2 (Cont.)

| **Depth** | **Age** | **δ^18^O** | **δ^13^C** | **Depth** | **Age** | **δ^18^O** | **δ^13^C** | **Depth** | **Age** | **δ^18^O** | **δ^13^C** |
| --- | --- | --- | --- | --- | --- | --- | --- | --- | --- | --- | --- |
| **(mm)** | **(CE)** | **(VPDB, ‰)** | **(VPDB, ‰)** | **(mm)** | **(CE)** | **(VPDB, ‰)** | **(VPDB, ‰)** | **(mm)** | **(CE)** | **(VPDB, ‰)** | **(VPDB, ‰)** |
| 38.21 | 1768.8 | -7.10 | -2.61 | 46.17 | 1715.0 | -7.15 | -1.87 | 54.91 | 1655.4 | -7.23 | -2.64 |
| 38.44 | 1767.5 | -7.15 | -2.30 | 46.31 | 1713.9 | -7.08 | -2.03 | 55.12 | 1654.3 | -7.11 | -2.11 |
| 38.66 | 1766.1 | -7.41 | -2.50 | 46.55 | 1712.0 | -7.07 | -1.69 | 55.32 | 1653.2 | -7.70 | -3.29 |
| 38.88 | 1764.7 | -7.31 | -2.14 | 46.80 | 1710.2 | -7.00 | -1.64 | 55.52 | 1652.2 | -7.38 | -2.87 |
| 39.11 | 1763.3 | -7.18 | -2.14 | 47.04 | 1708.3 | -6.94 | -1.51 | 55.73 | 1651.2 | -7.51 | -3.36 |
| 39.33 | 1762.0 | -7.14 | -1.82 | 47.28 | 1706.4 | -7.07 | -1.59 | 55.93 | 1650.1 | -7.32 | -2.81 |
| 39.55 | 1760.6 | -7.24 | -2.01 | 47.52 | 1704.6 | -6.90 | -1.34 | 56.13 | 1649.1 | -7.30 | -2.40 |
| 39.78 | 1759.2 | -7.08 | -1.80 | 47.77 | 1702.9 | -7.24 | -2.18 | 56.33 | 1648.1 | -7.55 | -2.43 |
| 40.00 | 1757.9 | -7.24 | -2.10 | 48.01 | 1701.2 | -7.15 | -2.81 | 56.53 | 1647.0 | -7.35 | -3.06 |
| 40.22 | 1756.5 | -7.06 | -1.70 | 48.25 | 1699.6 | -7.12 | -3.12 | 56.73 | 1646.0 | -7.31 | -2.86 |
| 40.44 | 1755.2 | -7.22 | -1.99 | 48.50 | 1697.9 | -7.09 | -2.75 | 56.94 | 1645.0 | -7.32 | -3.20 |
| 40.66 | 1753.8 | -7.00 | -1.62 | 48.74 | 1696.2 | -7.09 | -2.69 | 57.14 | 1643.9 | -7.40 | -3.23 |
| 40.88 | 1752.4 | -7.12 | -1.87 | 48.98 | 1694.5 | -7.10 | -2.45 | 57.34 | 1642.9 | -7.15 | -3.10 |
| 41.11 | 1751.1 | -6.88 | -1.78 | 49.22 | 1692.9 | -7.22 | -3.02 | 57.54 | 1641.9 | -7.28 | -3.05 |
| 41.33 | 1749.7 | -7.05 | -2.47 | 49.47 | 1691.2 | -7.08 | -2.32 | 57.74 | 1640.8 | -7.23 | -2.90 |
| 41.55 | 1748.4 | -7.21 | -2.34 | 49.71 | 1689.5 | -7.12 | -2.11 | 57.94 | 1639.8 | -7.17 | -2.87 |
| 41.77 | 1747.0 | -7.09 | -2.28 | 49.95 | 1687.9 | -7.04 | -1.75 | 58.15 | 1638.8 | -7.30 | -3.16 |
| 41.99 | 1745.7 | -7.16 | -1.89 | 50.19 | 1686.2 | -7.04 | -2.47 | 58.35 | 1637.7 | -7.45 | -2.81 |
| 42.21 | 1744.3 | -7.20 | -3.17 | 50.42 | 1684.6 | -7.24 | -2.35 | 58.55 | 1636.7 | -7.32 | -3.13 |
| 42.43 | 1742.9 | -7.16 | -2.79 | 50.66 | 1683.0 | -7.29 | -3.03 | 58.75 | 1635.7 | -7.31 | -2.83 |
| 42.65 | 1741.6 | -7.24 | -3.31 | 50.90 | 1681.4 | -7.33 | -2.52 | 58.95 | 1634.6 | -7.20 | -2.97 |
| 42.88 | 1740.2 | -7.38 | -3.03 | 51.13 | 1679.7 | -7.42 | -2.54 | 59.15 | 1633.6 | -7.18 | -2.07 |
| 43.10 | 1738.7 | -7.30 | -3.31 | 51.37 | 1678.1 | -7.35 | -2.20 | 59.35 | 1632.6 | -7.14 | -1.43 |
| 43.32 | 1737.0 | -7.03 | -2.69 | 51.60 | 1676.5 | -7.30 | -2.55 | 59.56 | 1631.5 | -7.41 | -1.87 |
| 43.54 | 1735.3 | -7.08 | -2.89 | 51.84 | 1674.9 | -7.29 | -2.53 | 59.76 | 1630.5 | -7.22 | -2.40 |
| 43.76 | 1733.6 | -7.10 | -2.71 | 52.08 | 1673.2 | -7.33 | -2.74 | 59.96 | 1629.4 | -7.64 | -1.73 |
| 43.98 | 1731.9 | -7.18 | -3.24 | 52.31 | 1671.6 | -7.03 | -2.29 | 60.20 | 1628.2 | -7.15 | -2.21 |
| 44.20 | 1730.2 | -7.07 | -2.13 | 52.55 | 1670.0 | -7.14 | -2.25 | 60.44 | 1627.0 | -7.30 | -2.25 |
| 44.42 | 1728.5 | -7.32 | -2.10 | 52.78 | 1668.4 | -6.93 | -2.14 | 60.69 | 1625.7 | -7.35 | -2.88 |
| 44.65 | 1726.8 | -7.14 | -1.76 | 53.02 | 1666.8 | -7.24 | -2.78 | 60.93 | 1624.5 | -7.45 | -2.79 |
| 44.87 | 1725.1 | -7.15 | -1.94 | 53.25 | 1665.1 | -7.10 | -2.44 | 61.18 | 1623.2 | -7.44 | -3.33 |
| 45.10 | 1723.3 | -7.48 | -2.30 | 53.49 | 1663.5 | -7.51 | -2.94 | 61.42 | 1622.0 | -7.38 | -2.84 |
| 45.34 | 1721.4 | -7.14 | -2.03 | 53.73 | 1661.9 | -7.47 | -2.96 | 61.67 | 1620.7 | -7.43 | -2.90 |
| 45.58 | 1719.5 | -7.13 | -2.12 | 53.96 | 1660.3 | -7.25 | -2.74 | 61.91 | 1619.4 | -7.41 | -2.67 |
| 45.68 | 1718.8 | -7.06 | -2.23 | 54.20 | 1659.0 | -7.08 | -2.32 | 62.16 | 1618.2 | -7.25 | -3.00 |
| 45.83 | 1717.7 | -7.18 | -1.90 | 54.43 | 1657.8 | -7.47 | -3.60 | 62.40 | 1616.9 | -7.07 | -2.07 |
| 46.07 | 1715.8 | -7.17 | -1.75 | 54.67 | 1656.6 | -7.12 | -2.64 | 62.65 | 1615.7 | -7.37 | -2.67 |

Continue to next page

Table S2 (Cont.)

| **Depth** | **Age** | **δ^18^O** | **δ^13^C** | **Depth** | **Age** | **δ^18^O** | **δ^13^C** | **Depth** | **Age** | **δ^18^O** | **δ^13^C** |
| --- | --- | --- | --- | --- | --- | --- | --- | --- | --- | --- | --- |
| **(mm)** | **(CE)** | **(VPDB, ‰)** | **(VPDB, ‰)** | **(mm)** | **(CE)** | **(VPDB, ‰)** | **(VPDB, ‰)** | **(mm)** | **(CE)** | **(VPDB, ‰)** | **(VPDB, ‰)** |
| 62.89 | 1614.4 | -7.34 | -2.75 | 71.61 | 1564.9 | -7.20 | -2.73 | 79.34 | 1504.7 | -7.03 | -2.11 |
| 63.14 | 1613.2 | -7.36 | -3.06 | 71.82 | 1563.5 | -7.26 | -2.25 | 79.42 | 1503.6 | -7.22 | -2.04 |
| 63.38 | 1611.9 | -7.14 | -2.73 | 72.02 | 1562.2 | -7.18 | -2.47 | 79.56 | 1501.9 | -7.09 | -2.03 |
| 63.63 | 1610.6 | -7.34 | -3.00 | 72.23 | 1560.8 | -7.36 | -2.38 | 79.65 | 1500.8 | -7.09 | -2.17 |
| 63.87 | 1609.4 | -7.21 | -2.89 | 72.44 | 1559.4 | -7.38 | -2.55 | 79.78 | 1499.2 | -7.01 | -2.36 |
| 64.12 | 1608.1 | -7.35 | -2.95 | 72.64 | 1557.6 | -7.15 | -2.53 | 79.87 | 1498.0 | -7.13 | -2.67 |
| 64.36 | 1606.9 | -7.33 | -2.58 | 72.85 | 1555.6 | -7.39 | -2.98 | 80.00 | 1496.4 | -7.07 | -2.47 |
| 64.61 | 1605.6 | -7.25 | -2.76 | 73.06 | 1553.6 | -7.30 | -2.48 | 80.10 | 1495.1 | -7.17 | -2.34 |
| 64.85 | 1604.4 | -7.15 | -2.45 | 73.26 | 1551.5 | -7.57 | -2.98 | 80.26 | 1493.2 | -7.12 | -1.69 |
| 65.10 | 1603.1 | -7.24 | -2.76 | 73.47 | 1549.5 | -7.74 | -2.98 | 80.36 | 1491.9 | -7.17 | -1.49 |
| 65.34 | 1601.9 | -7.43 | -2.60 | 73.68 | 1547.5 | -7.63 | -3.15 | 80.52 | 1490.0 | -7.11 | -1.36 |
| 65.58 | 1600.6 | -7.28 | -2.83 | 73.88 | 1545.5 | -7.71 | -2.79 | 80.62 | 1488.7 | -7.00 | -1.43 |
| 65.83 | 1599.4 | -7.14 | -2.48 | 74.09 | 1543.5 | -7.57 | -3.02 | 80.77 | 1486.7 | -6.97 | -1.36 |
| 66.07 | 1598.1 | -7.25 | -2.74 | 74.30 | 1541.4 | -7.41 | -2.65 | 80.88 | 1485.5 |  |  |
| 66.31 | 1596.9 | -7.23 | -2.34 | 74.50 | 1539.4 | -7.37 | -2.41 | 81.03 | 1483.5 | -7.12 | -1.47 |
| 66.55 | 1595.6 | -7.25 | -1.84 | 74.71 | 1537.4 | -7.32 | -2.39 | 81.13 | 1482.2 | -7.13 | -1.43 |
| 66.80 | 1594.4 | -7.03 | -0.95 | 74.92 | 1535.4 | -7.25 | -2.54 | 81.29 | 1480.3 | -7.11 | -1.34 |
| 67.04 | 1593.2 | -7.08 | -2.04 | 75.13 | 1533.8 | -7.17 | -2.46 | 81.29 | 1480.3 | -7.17 | -1.41 |
| 67.28 | 1591.9 | -7.12 | -2.35 | 75.35 | 1532.5 | -7.39 | -2.82 | 81.55 | 1477.1 | -7.12 | -1.30 |
| 67.52 | 1590.7 | -7.22 | -2.59 | 75.58 | 1531.1 | -7.05 | -2.41 | 81.65 | 1475.8 | -7.01 | -1.31 |
| 67.77 | 1589.4 | -7.32 | -2.53 | 75.80 | 1529.8 | -7.04 | -2.68 | 81.80 | 1473.9 | -6.99 | -1.37 |
| 68.01 | 1588.2 | -7.38 | -2.71 | 76.02 | 1528.5 | -7.12 | -2.48 | 81.91 | 1472.6 | -7.03 | -1.45 |
| 68.25 | 1586.9 | -7.17 | -2.14 | 76.24 | 1527.1 | -6.88 | -2.75 | 82.06 | 1470.6 | -6.95 | -1.42 |
| 68.50 | 1585.7 | -7.33 | -2.29 | 76.46 | 1525.8 | -6.96 | -2.77 | 82.16 | 1469.4 | -6.96 | -1.41 |
| 68.74 | 1584.1 | -7.10 | -2.49 | 76.68 | 1524.5 | -7.07 | -2.73 | 82.32 | 1467.4 | -7.01 | -1.34 |
| 68.98 | 1582.5 | -7.44 | -3.09 | 76.90 | 1523.1 | -7.28 | -2.89 | 82.42 | 1466.1 | -7.08 | -1.41 |
| 69.22 | 1580.8 | -7.63 | -2.90 | 77.12 | 1521.8 | -7.28 | -2.99 | 82.58 | 1464.2 | -7.00 | -1.27 |
| 69.47 | 1579.2 | -7.56 | -3.02 | 77.35 | 1520.5 | -7.45 | -2.93 | 82.68 | 1462.9 | -7.06 | -1.45 |
| 69.71 | 1577.6 | -7.44 | -2.56 | 77.57 | 1519.1 | -7.33 | -2.99 | 82.84 | 1461.0 | -6.82 | -1.38 |
| 69.95 | 1576.0 | -7.34 | -2.85 | 77.79 | 1517.8 | -7.14 | -2.66 | 82.94 | 1459.7 | -7.16 | -1.71 |
| 70.17 | 1574.6 | -7.23 | -2.67 | 78.01 | 1516.5 | -7.14 | -2.28 | 83.09 | 1457.8 | -7.07 | -1.60 |
| 70.37 | 1573.2 | -7.23 | -2.92 | 78.23 | 1515.1 | -7.10 | -1.87 | 83.20 | 1456.5 | -7.08 | -1.28 |
| 70.58 | 1571.8 | -7.38 | -2.84 | 78.45 | 1513.8 | -7.05 | -2.52 | 83.35 | 1454.6 | -7.11 | -1.92 |
| 70.79 | 1570.4 | -7.37 | -3.06 | 78.67 | 1512.5 | -7.27 | -2.43 | 83.45 | 1453.3 | -7.16 | -2.29 |
| 70.99 | 1569.1 | -7.40 | -2.67 | 78.89 | 1510.2 | -7.11 | -2.50 | 83.61 | 1451.3 | -7.18 | -1.95 |
| 71.20 | 1567.7 | -7.38 | -2.81 | 79.12 | 1507.4 | -7.13 | -2.46 | 83.71 | 1450.1 | -7.17 | -1.80 |
| 71.40 | 1566.3 | -7.28 | -2.51 | 79.20 | 1506.3 | -7.23 | -2.46 | 83.87 | 1448.1 | -7.07 | -2.06 |

Continue to next page

Table S2 (Cont.)

| **Depth** | **Age** | **δ^18^O** | **δ^13^C** | **Depth** | **Age** | **δ^18^O** | **δ^13^C** | **Depth** | **Age** | **δ^18^O** | **δ^13^C** |
| --- | --- | --- | --- | --- | --- | --- | --- | --- | --- | --- | --- |
| **(mm)** | **(CE)** | **(VPDB, ‰)** | **(VPDB, ‰)** | **(mm)** | **(CE)** | **(VPDB, ‰)** | **(VPDB, ‰)** | **(mm)** | **(CE)** | **(VPDB, ‰)** | **(VPDB, ‰)** |
| 83.97 | 1446.8 | -7.24 | -2.37 | 88.79 | 1385.0 | -7.17 | -2.80 | 93.43 | 1322.6 | -6.81 | -1.19 |
| 84.12 | 1444.9 | -7.05 | -2.27 | 88.88 | 1383.7 | -7.16 | -2.84 | 93.53 | 1321.3 | -6.86 | -1.05 |
| 84.23 | 1443.6 | -7.24 | -2.35 | 89.03 | 1381.7 | -6.97 | -2.75 | 93.68 | 1319.3 | -6.89 | -2.21 |
| 84.38 | 1441.7 | -7.07 | -1.61 | 89.13 | 1380.4 | -7.07 | -2.74 | 93.77 | 1318.0 | -6.95 | -2.39 |
| 84.48 | 1440.4 | -7.11 | -1.43 | 89.27 | 1378.5 | -6.87 | -2.50 | 93.92 | 1316.0 | -7.00 | -2.63 |
| 84.64 | 1438.5 | -7.16 | -1.43 | 89.37 | 1377.2 | -7.28 | -2.42 | 94.02 | 1314.7 | -7.04 | -2.46 |
| 84.74 | 1437.2 | -7.02 | -1.40 | 89.51 | 1375.2 | -7.04 | -2.79 | 94.17 | 1312.7 | -6.84 | -2.37 |
| 84.90 | 1435.2 | -7.11 | -1.19 | 89.61 | 1373.9 | -7.15 | -2.66 | 94.26 | 1311.4 | -6.97 | -2.58 |
| 85.00 | 1434.0 | -6.96 | -1.36 | 89.76 | 1371.9 | -7.34 | -2.73 | 94.41 | 1309.4 | -7.05 | -2.69 |
| 85.15 | 1432.1 | -7.00 | -1.36 | 89.85 | 1370.6 | -7.14 | -2.70 | 94.51 | 1308.1 | -7.12 | -2.86 |
| 85.24 | 1430.9 | -7.06 | -1.37 | 90.00 | 1368.7 | -7.10 | -2.54 | 94.66 | 1306.1 | -7.05 | -2.49 |
| 85.39 | 1429.1 | -6.99 | -1.51 | 90.10 | 1367.4 | -7.27 | -2.57 | 94.75 | 1304.8 | -7.01 | -1.90 |
| 85.49 | 1427.9 | -7.12 | -1.44 | 90.25 | 1365.4 | -7.07 | -2.71 | 94.90 | 1302.8 | -7.06 | -1.73 |
| 85.63 | 1426.1 | -7.09 | -1.62 | 90.34 | 1364.1 | -6.97 | -2.42 | 95.00 | 1301.5 | -6.97 | -1.65 |
| 85.73 | 1424.9 | -6.96 | -2.15 | 90.49 | 1362.1 | -6.88 | -1.99 | 95.14 | 1299.6 | -6.94 | -2.34 |
| 85.87 | 1423.1 | -6.88 | -1.76 | 90.59 | 1360.8 | -6.91 | -1.97 | 95.24 | 1298.3 | -6.96 | -2.31 |
| 85.97 | 1421.8 | -6.87 | -1.66 | 90.74 | 1358.8 | -7.01 | -2.44 | 95.38 | 1296.3 | -6.90 | -2.17 |
| 86.12 | 1420.0 | -6.93 | -1.21 | 90.83 | 1357.5 | -7.09 | -2.08 | 95.48 | 1295.1 | -7.10 | -2.23 |
| 86.21 | 1418.8 | -6.93 | -1.25 | 90.98 | 1355.5 | -7.27 | -2.79 | 95.63 | 1293.1 | -7.05 | -1.81 |
| 86.36 | 1417.0 | -6.91 | -1.67 | 91.08 | 1354.2 | -7.16 | -2.69 | 95.72 | 1291.8 | -6.98 | -1.63 |
| 86.46 | 1415.8 | -7.29 | -2.31 | 91.23 | 1352.2 | -6.98 | -2.60 | 95.87 | 1289.9 | -7.01 | -1.57 |
| 86.60 | 1414.0 | -6.98 | -1.98 | 91.32 | 1350.9 |  |  | 95.96 | 1288.6 | -7.01 | -1.61 |
| 86.70 | 1412.7 | -6.89 | -1.80 | 91.47 | 1348.9 | -7.00 | -2.55 | 96.11 | 1286.7 | -7.02 | -1.76 |
| 86.84 | 1410.9 | -7.02 | -1.89 | 91.57 | 1347.6 | -7.06 | -2.57 | 96.20 | 1285.4 | -7.02 | -2.23 |
| 86.94 | 1409.7 | -6.88 | -2.14 | 91.72 | 1345.6 | -7.08 | -2.56 | 96.35 | 1283.4 | -7.07 | -2.47 |
| 87.09 | 1407.8 | -6.86 | -1.96 | 91.81 | 1344.3 | -7.25 | -2.55 | 96.44 | 1282.1 | -7.25 | -2.52 |
| 87.18 | 1406.5 | -7.04 | -1.92 | 91.96 | 1342.3 | -7.06 | -2.59 | 96.59 | 1280.2 | -6.81 | -2.15 |
| 87.33 | 1404.6 | -6.91 | -1.95 | 92.06 | 1341.0 | -7.18 | -2.74 | 96.83 | 1277.6 | -6.92 | -2.52 |
| 87.43 | 1403.2 | -6.96 | -1.96 | 92.21 | 1339.0 | -6.93 | -2.53 | 97.07 | 1276.2 | -6.93 | -2.39 |
| 87.57 | 1401.3 | -6.97 | -2.11 | 92.30 | 1337.7 | -7.05 | -2.41 | 97.31 | 1274.9 | -7.00 | -2.22 |
| 87.82 | 1398.0 | -6.95 | -2.46 | 92.45 | 1335.8 | -6.97 | -2.30 | 97.55 | 1273.5 | -6.76 | -2.11 |
| 87.91 | 1396.7 | -7.06 | -2.36 | 92.55 | 1334.4 | -7.10 | -2.26 | 97.79 | 1272.2 | -6.94 | -2.43 |
| 88.06 | 1394.8 | -6.97 | -2.30 | 92.70 | 1332.5 | -6.98 | -2.43 | 98.03 | 1270.8 | -7.09 | -2.66 |
| 88.16 | 1393.5 | -7.08 | -2.30 | 92.79 | 1331.1 | -7.10 | -2.66 | 98.27 | 1269.5 | -7.05 | -1.75 |
| 88.30 | 1391.5 | -7.10 | -2.62 | 92.94 | 1329.2 | -7.19 | -2.59 | 98.51 | 1268.1 | -6.72 | -1.66 |
| 88.40 | 1390.2 | -7.22 | -2.99 | 93.04 | 1327.9 | -7.13 | -2.45 | 98.75 | 1266.8 | -6.80 | -2.13 |
| 88.54 | 1388.2 | -6.81 | -2.16 | 93.19 | 1325.9 | -6.95 | -1.74 | 98.99 | 1265.5 | -6.96 | -1.52 |
| 88.64 | 1386.9 | -7.33 | -2.72 | 93.28 | 1324.6 | -6.95 | -1.31 |  |  |  |  |
